# Supplementary material for: Clinical Phenotypes of COVID-19 Associated Mucormycosis (CAM): A Comprehensive Review
Source: Diagnostics (Basel). 2022 Dec 8;12(12):3092. doi: 10.3390/diagnostics12123092 (PMC9777018; doi:10.3390/diagnostics12123092)
Supplement: Supplementary file 1 [file diagnostics-12-03092-s001.zip › diagnostics-2057109-supplementary.pdf]

Table S1: Interval between CAM diagnosis and COVID-19 diagnosis and other comorbidities described

Study	Days after diagnosis	Other comorbidities (% of CAM pts)
Said Ahmed WM et al. [29]	14–30 post recovery	NA
Murthy R et al. [30]	NA	NA
Walia S et al. [31]	22.56 ± 4.78	Remdesivir (28.89%)<br>Oxygen treatment (52.40%)
Vare AA et al. [32]	15 post recovery	NIV or MV (27%),<br>Prolonged oxygen treatment (78%)
Fouad YA et al. [33]	20.5	HTN (26.9%),<br>CKD (11.5%),<br>IHD (7.7%),<br>Bronchial Asthma (7.7%),<br>Liver disease (3.8%)
Soni K te al. [34]	NA	HIV (0.7%),<br>Renal transplant (0.7%),<br>Hematological Malignancy (1.38%),<br>Heart Disease (39.3%)
Metwally MI et al. [35]	3 weeks	HTN (41.2%),<br>Chronic Liver Disease (11.1%),<br>Heart Failure (7.9%),<br>COPD (6.3%),<br>Bronchial Asthma (3.1%),<br>Thyrotoxicosis (1.5%),<br>Multiple Myeloma (1.5%)
Arora U et al. [36]	18.9 (SD 9.1)	Significant association with CAM : Number of times swab testing,
Jindal G et al. [37]	NA	HTN (33%)
Syed-Abdul S et al. [38]	NA	NA
Patel A et al. [3]	NA	NA
Pruthi H et al. [39]	23.6	NA
Bansal SB et al. [40]	17	NA
Dulski TM et al. [41]	18.5	SOT (10%)
Meshram HS et al. [42]	NA	CKD (8.2%),<br>HTN (31.1%),<br>IgA Nephropathy (3.3%),<br>Renal stone disease (8.2%),<br>Chronic Glomerulonephritis (18%), Autosomal polycystic kidney (3.3%)
Aggarwal SK et al. [43]	NA	HTN (38.4%)
Kulkarni R et al. [44]	18	HTN (36.7%)
Choksi T et al. [45]	28	NA
Kumar S et al. [46].	10-20	NA

Mehta R et al. [47]	2-5 weeks post recovery	HTN (35.3%)
Panwar P et al. [48]	11.4	NA
Patel DD et al. [49]	NA	HTN (39.6%),<br>IHD (6.2%)
Vaid N et al. [50]	NA	NA
Goddanti N et al. [51]	NA	Oxygen Treatment (60%)
Yadav T et al. [52]	14.2 ± 7.12	NA
Meshram VB et al. [53]	7 post recovery	HTN (72.7%)
Zirpe K et al. [54]	11	HTN (36.9%)
Alloush TK et al. [55]	12.5	HTN (42,8%),<br>CKD (14.2%),<br>IHD (7.1%)
Pal P et al. [56]	22.9	NA
Danion F et al. [57]	24	SOT<br>Hematological Malignancies (41%),<br>Renal Failure (65%)
Nehara HR et al. [58]	15	Hematologic malignancy (1.9%)
Pandiar D et al. [59]	NA	NA
Kumar S et al. [60]	NA	Zinc (89.1%),<br>HTN 31/55 (56.4%),
Bilgic A et al. [61]	17.5	HTN (13%),<br>Hypothyroidism (10.5%),<br>Renal Dysfunction (2.6%),<br>Rheumatoid Arthritis (2.6%)
Guemas E et al. [62]	4.5	NA
Kumar SG et al. [63]	NA	NA
Mani S et al. [64]	17.36 ± 7.3	NA
Dravid A et al. [65]	17	HTN (40.7%),<br>CKD (15.3%),<br>IHD(11.9%),<br>Chronic liver disease (3.4%)
Naruka S et al. [66]	NA	NA
Jain K et al. [67]	20	NA
Bhanuprasad K et al. [68]	NA	Chronic lung disease (2.3%),<br>IHD (3.8%),<br>CKD (5.3%),<br>Cerebrovascular events (1.5%),<br>HIV (1.5%)
Desai EJ et al. [69]	NA	HTN (33%),<br>IHD (9%),<br>Hypothyroidism (2%),<br>CKD (4%)
Nasir \n et al. [70]	16	Haematological malignancies (20%), Tocilizumab treatment (20%)
Gupta\s et al. [71]	NA	HTN (27%)

Joshi S et al. [4]	28	HIV (0.56%),<br>HTN (27%),<br>CKD (3%),<br>Cardiomyopathy (2%),<br>Bronchial Asthma (1%)
Pradhan P et al. [72]	NA	NA
Mehta RNM et al. [73]	NA	NA
Riad A et al. [74]	NA	NA
Guzmán-Castro S et al. [75]	15	NA
Seidel D et al. [76]	10	Immunosuppresion (38.46%)
Gupta R et al. [77]	NA	IHD (15.6%),<br>CKD (10.4%),<br>Renal transplant (2.6%)
Alfishawy M et al. [78]	NA	NA
Dave TV et al. [79]	16 ± 21	NA
Selarka L et al. [80]	12.1 ± 4.6	HTN (57.4%),<br>IHD (12.7%),<br>COPD (4.3%)
Avatef Fazeli M et al. [81]	25.66 ± 12.84	HTN (58.33%)<br>IHD (33.33%)<br>CKD (16.66%)
Mishra Y et al. [82]	NA	HTN (50%),<br>IHD (6.3%)
Sen M et al. [83]	14.5 ± 10	HTN (80%),<br>Renal disease (10%),<br>Bronchial Asthma (2%)
Pakdel F et al. [84]	7 (range: 1-37)	NA
Y M. Reddy et al. [85]	NA	Renal dysfunction
R.Arora et al. [86]	17	HTN, IHD, CKD, thyroid dysfunction
D.P Gupta et al. [87]	NA	Renal transplant, acute myeloid leukemia
M.Gautam et al. [88]	NA	NA
R.M.Mehta et al. [89]	25.8	HTN, CKD, IHD
Y.M.Reddy et al. [90]	16.28 ±6.6	HTN, CKD, IHD, hypothyroidism, asthma, epilepsy, stroke
S.P.Singh et al. [91]	15.3	NA
M.Hada et al. [92]	12.4	NA
M. Kumar H et al. [93]	NA	NA
S. Bhandari et al. [94]	Within 45 days	HTN, CKD, malignancy
M Chouhan et al. [95]	2-48	CKD
Y. Singh et al. [96]	NA	HTN, renal transplant, hypothyroidism, asthma, malignancy, IHD
S M Desai et al. [97]	10 days – 5 months	HTN, malignancy
A. Kumari et al. [98]	NA	CKD, liver disease
S. Mitra et al. [99]	18 ± 4	HTN, CKD
A Ramaswami et al. [100]	20	HTN, IHD, SOT, CKD

A.R. Joshi et al. [101]	NA	HIV
A. Patel et al. [102]	18	Hematological malignancy, Renal transplant
S Sharma et al. [103]	NA	HTN, renal failure
R. Kant et al. [104]	NA	HTN, CKD, COPD
C. Eker et al. [105]	30.3 ± 26.6	NA
A.K. Pandit et al. [106]	4	HTN, obesity
S.F. Youssif et al. [107]	NA	HTN, CKD, liver disease, cardiac disease
A. Sekaran, et al. [108]	NA	HTN, AKI
R. R. Shabana et al. [109]	2.8 weeks post recovery	NA
A. K Patel et al. [110]	23.4 ± 9.3	Cardiovascular disease, lung disease, CKD
H. D.D. Martins et al. [111]	NA	HTN, non-Hodgkin lymphoma
S. Iqtadar et al. [112]	NA	HTN, CKD, asthma
A. Al Balushi et al. [113]	10d-3 w	NA
R. Soman et al. [114]	NA	HTN, CKD

AKI: Acute kidney Injury, CKD: chronic kidney disease, COPD: chronic obstructive pulmonary disease, IHD: ischemic heart disease, HTN: hypertension, MV : Mechanical Ventilation, NA : Not Available, NIV: Non Invasive Ventilation, SOT : Solid Organ Transplantation

Table S2: Cases of pulmonary mucormycosis associated with COVID-19

Study	n	MV	CAPA	Mortality
Walia S et al. [31]	1	NA	NA	NA
Arora U et al. [36]	2	NA	NA	NA
Patel A et al. [3]	1	NA	NA	NA
Pruthi H et al [39]	5	0/5 (0%)	NA	4/5 (80%)
Bansal SB et al. [40]	1	0/1 (0%)	NA	0/1 (0%)
Dulski TM et al. [41]	3	NA	NA	NA
Meshram HS et al. [42]	5	0/5 (0%)	NA	5/5 (100%)
Meshram VB et al. [53]	1	0/1 (0%)	NA	1/1 (100%)
Danion F et al. [57]	9	7/9 (77.8%)	2/9 (22.2%)	8/9 (88.9%)
Guemas E et al. [62]	10	ICU pts	8/10 (80%)	5/10 (50%)
Nasir \n et al. [70]	6	3/6 (50%)	3/6 (50%)	6/6 (100%)
Guzmán-Castro S et al. [75]	1	1/1 (100%)	0/1 (0%)	1/1 (100%)
Seidel D et al. [76]	11	10/11 (90.9%)	2/11 (18.2%)	6/11 (54.5%)

Alfishawy M et al. [78]	1	NA	1/1 (100%)	0/1 (0%)
R.M.Mehta et al [89]	5	4/5 (80%)	3/5 (60%)	4/5 (80%)
M. Kumar H et al. [93]	4	NA	NA	2/4 (50%)
Y. Singh et al. [96]	1	1/1 (100%)	NA	1/1 (100%)
A. Patel et al. [102]	16	NA	NA	NA
R. Kant et al. [104]	4	NA	NA	NA
A. K Patel et al. [110]	5	NA	NA	NA
R. Soman et al. [114]	6	NA	NA	NA

MV: mechanical ventilation, NA: not available, CAPA: COVID-19 Associated Pulmonary Aspergillosis

Table S3: Studies containing data on PCR and species identification

Study	PCR	Mucorales species
Patel A et al [3]	NA	*Rhizopus arrhizus* (8/29),<br>*Cunninghamella bertholletiae* (1/29)
Pruthi H et al [39]	2/5	*Rhizopus arrhizus* (2/5),<br>*Rhizopus homothallicus* (1/5),<br>*Rhizopus microspores* (1/5)
Meshram HS et al [42]	NA	*Rhizopus* spp (18/61),<br>*Rhizomucor* (4/61)
Aggarwal SK et al [43]	NA	*Mucor* spp,<br>*Rhizopus* spp
Danion F et a. [57]	Positive: 15/17 (88%)<br>Serum (14/17)<br>BAL (7/17)<br>Tissues (3/17)<br>Peritoneal fluid (1/17])	*Rhizopus microsporus* (6/17),<br>*Rhizopus delamar* (2/17),<br>*Rhizopus arrhizus* (1/17),<br>*Rhizomucor pusillus* (1/17),<br>*Rhizomucor miehei* (1/17),<br>*Lichtheimia* spp (1/17)
Bilgic A et al [61]	NA	*Rhizopus* spp (23/38),<br>*Mucor* spp (8/38),<br>*Absidia* (7/38)
Guemas E et al [62]	10/141 respiratory samples	*Mucor*<br>*Rhizopus*
Nasir N et al [70]	NA	*Rhizopus* spp (7/10),<br>*Mucor* spp (2/10)
Mehta NMR et al [73]	NA	*Rhizopus* spp (170/215)<br>*Lichtemia* (28/215)<br>*Apophysomyces* (11/215)
Riad A et al [74]	2/7 (28.5%)	NA
Guzmán-Castro S et al [75]	NA	*Mucor circinelloides* (3/4),<br>*Rhizopus pucilus* (1/4)
Seidel D et al [76]	NA	*Rhizopus* spp. (10/13),

		R 6icrospores (7/13),<br>*Lichtheimia* (2/13),<br>*Rhizomucor* (1/13)
Alfishawy M et al [78]	NA	*Mucorales* (3/21),<br>*Rhizopus* spp (1/21)
R.M.Mehta et al. [89]	2/4	*Rhizopous delemar (1/5),*<br>*Rhizopus oryzae (1/5)*
R. Kant et al. [104]	NA	*Rhizopus arrhizus (36/76), R. Arrihizus (1/76)*
A. K Patel et al. [110]	NA	*Rhizopus arrhizus (23/64), Rhizopus 6icrospores (3/64), Rhizopus species(4/64), Cunninghamella (1/64), Mucorales (1/64)*
A. Al Balushi et al. [113]	NA	*Rhizopus oryzae (8/10)*

NA: not available

Table S4: Concomitant *Aspergillus* isolation.

**Study**	**Concomitant *Aspergillus* isolation, n (%)**
Danion F et al. [57]	5/17 (29.4%)
Guemas E et al. [62]	8/10 (80%)
Dravid A et al. [65]	3/59 (5.1%)
Naruka S et al. [66]	16/79 (20.3%)
Jain K et al. [67]	1/95 (1.1%)
Nasir \n et al. [70]	4/10 (40%)
Seidel D et al. [76]	2/13 (15.4%)
Alfishawy M et al. [78]	4/21 (19%)
Selarka L et al. [80]	10/47 (21.3%)
R. Arora et al. [86]	2/37 (5.4%)
R.M.Mehta et al [89]	3/5 (60%)
S M Desai et al. [97]	3/50 (6%)
S. Mitra et al. [99]	1/32 (3.1%)
A. Patel et al. [102]	1/187 (0.5%)
R. Kant et al. [104]	14/100 (14%)
A. Sekaran, et al. [108]	1/30 (3.3%)
A. K Patel et al. [110]	9/64 (14.1%)
S. Iqtadar et al. [112]	2/7 (28.6%)

Table S5: Outcome

Study	CAM patients (n)	CAM patients that died (n)	Mortality (%)	Risk factors associated with mortality
Said Ahmed WM et al. [29]	14	NA	NA	NA
Murthy R et al. [30]	NA	NA	NA	NA
Walia S et al. [31]	540	50	9.25%	NA
Vare AA et al. [32]	67	23	34%	NA
Fouad YA et al. [33]	26	12	46,2%	NA
Soni K te al. [34]	145	26	18%	NA
Metwally MI et al. [35]	63	11	17.5%	NA
Arora U et al. [36]	152	NA	NA	NA
Jindal G et al. [37]	15	1	6.6%	NA
Syed-Abdul S et al. [38]	214	NA	NA	NA
Patel A et al. [3]	29	NA	NA	NA
Pruthi H et al. [39]	5	4	80%	NA
Bansal SB et al. [40]	11	2	18.2%	NA
Dulski TM et al. [41]	10	6	60%	NA
Meshram HS et al. [42]	61	16	26.2%	Older age, Obesity, Difficulty of breathing, High-flow oxygen requirement, delay in starting therapy
Aggarwal SK et al. [43]	13	2	15.4%	NA
Kulkarni R et al. [44]	49	25	51%.	NA
Choksi T et al. [45]	73	26	36%	NIV/MV, uncontrolled diabetes, HRCT score during COVID 19
Kumar S et al. [46].	287	NA	NA	NA
Mehta R et al. [47]	17	NA	NA	NA
Panwar P et al. [48]	7	0	0%	NA
Patel DD et al. [49]	NA	NA	NA	NA
Vaid N et al. [50]	65	7	10.7%	Intracranial involvement
Goddanti N et al. [51]	300	NA	NA	NA
Yadav T et al. [52]	50	NA	NA	NA

Meshram VB et al. [53]	11	3	27%	NA
Zirpe K et al. [54]	84	13	15.5%	CKD, orbital involvement, tocilizumab, renal dysfunction
Alloush TK et al. [55]	14	3	21.4%	NA
Pal P et al. [56]	10	3	30%	NA
Danion F et al. [57]	17	15	88%	NA
Nehara HR et al. [58]	105	20	19.05%	ROCM stage≥3c, qSOFA≥2
Pandiar D et al. [59]	12	NA	NA	NA
Kumar S et al. [60]	55	9	16%	NA
Bilgic A et al. [61]	38	2	5%	NA
Guemas E et al. [62]	10	5	50%	NA
Kumar SG et al. [63]	101	18	17.8%	higher imaging stages
Mani S et al. [64]	89	3	3.4%	Stage ≥3c (involvement of orbit and CNS)
Dravid A et al. [65]	59	15	25.4%	CT severity index≥18, eye sweling, loss of vision, ptosis, limb weakness, diabetic ketoacidosis, cerebral involvement
Naruka S et al. [66]	79	14	18.18%	NA
Jain K et al. [67]	95	5	5.2%	Severity grade of histopathology.
Bhanuprasad K et al. [68]	132	13	9.8%	NA
Desai EJ et al. [69]	100	20	20%	NA
Nasir \n et al. [70]	10	7	70%	NA
Gupta\s et al. [71]	56	9	16%	NA
Joshi S et al. [4]	178	26	15%	NA
Pradhan P et al. [72]	46	9	19.5%	NA
Mehta RNM et al. [73]	215	26	12.1%	Cerebral disease
Riad A et al. [74]	7	0	0%	
Guzmán-Castro S et al. [75]	6	5	83.3%	NA
Seidel D et al. [76]	13	7	53.8%	NA
Gupta R et al. [77]	115	25	21.7%	CT scan-based score for severity of lung involvement
Alfishawy M et al. [78]	21	7	33.3%	NA
Dave TV et al. [79]	58	20	34%	CNS involvement
Selarka L et al. [80]	47	11	23.4%	NA
Avatef Fazeli M et al. [81]	12	8	66.7%	NA
Mishra Y et al. [82]	32	4	12.5%	NA
Sen M et al. [83]	2218	305	14%	NA
Pakdel F et al. [84]	15	7	47%	NA
Y M. Reddy et al. [85]	6	6	100%	NA
R.Arora et al. [86]	NA	NA	NA	NA
D.P Gupta et al. [87]	70	4	5.7%	NA
M.Gautam et al. [88]	30	0	0%	NA
R.M.Mehta et al. [89]	5	4	80%	NA
Y.M.Reddy et al. [90]	31	11	35.5%	NA
S.P.Singh et al. [91]	6	1	16.7%	NA

M.Hada et al. [92]	NA	NA	NA	NA
M. Kumar H et al. [93]	23	17	73.9%	NA
S. Bhandari et al. [94]	NA	NA	NA	NA
M Chouhan et al. [95]	41	4	9.8%	NA
Y. Singh et al. [96]	13	9	69.2%	NA
S M Desai et al. [97]	50	15	30%	NA
A. Kumari et al. [98]	20	6	30%	NA
S. Mitra et al. [99]	NA	NA	NA	NA
A Ramaswami et al. [100]	NA	NA	NA	NA
A.R. Joshi et al. [101]	25	14	56%	NA
A. Patel et al. [102]	170	75	44.1%	age, site of involvement (rhino-orbital-cerebral or pulmonary), and ICU admission
S Sharma et al. [103]	NA	NA	NA	NA
R. Kant et al. [104]	100	13	13%	NA
C. Eker et al. [105]	15	5	33.3%	NA
A.K. Pandit et al. [106]	49	15	30.6%	NA
S.F. Youssif et al. [107]	33	30	90.9%	NA
A. Sekaran, et al. [108]	30	5	16.7%	NA
R. R. Shabana et al. [109]	30	6	20%	NA
A. K Patel et al. [110]	64	3	4.7%	NA
H. D.D. Martins et al. [111]	6	1	16.7%	NA
S. Iqtadar et al. [112]	7	1	14.3%	NA
A. Al Balushi et al. [113]	10	6	60%	NA
R. Soman et al. [114]	28	7	25%	NA

HRCT : High-resolution computed tomography, qSOFA : quick Sequential Organ Failure Assessment, MV : Mechanical ventilation, NA: not available, NIV: non – invasive ventilation, CKD: chronic kidney disease, CNS: central nervous system, ROCM: rhino- orbital- cerebral mucormycosis

Table S6: Percentage of deaths with cerebral and pulmonary involvement.

Study	% (n) of deaths with cerebral involvement	% (n) of deaths with pulmonary involvement
Said Ahmed WM et al. [29]	-	-
Murthy R et al. [30]	-	-
Walia S et al. [31]	88% (44/50)	NA
Vare AA et al. [32]	NA	-
Fouad YA et al. [33]	-	-
Soni K te al. [34]	NA	-
Metwally MI et al. [35]	NA	-
Arora U et al. [36]	NA	NA
Jindal G et al. [37]	NA	-
Syed-Abdul S et al. [38]	NA	NA
Patel A et al. [3]	NA	NA
Pruthi H et al. [39]	-	100% (4/4)
Bansal SB et al. [40]	NA	0% (0/2)
Dulski TM et al. [41]	NA	NA
Meshram HS et al. [42]	NA	31.3% (5/16)
Aggarwal SK et al. [43]	50% (1/2)	-
Kulkarni R et al. [44]	NA	-
Choksi T et al. [45]	NA	-
Kumar S et al. [46].	NA	-
Mehta R et al. [47]	NA	-
Panwar P et al. [48]	-	-
Patel DD et al. [49]	NA	-
Vaid N et al. [50]	100% (7/7)	-
Goddanti N et al. [51]	NA	-
Yadav T et al. [52]	NA	-
Meshram VB et al. [53]	66.7% (2/3)	33.3% (1/3)
Zirpe K et al. [54]	38.5% (5/13)	-

Alloush TK et al. [55]	NA	-
Pal P et al. [56]	33.3% (1/3)	-
Danion F et al. [57]	NA	53.3% (8/15)
Nehara HR et al. [58]	NA	-
Pandiar D et al. [59]	NA	-
Kumar S et al. [60]	NA	-
Bilgic A et al. [61]	NA	-
Guemas E et al. [62]	-	100% (5/5)
Kumar SG et al. [63]	NA	-
Mani S et al. [64]	66.7% (2/3)	-
Dravid A et al. [65]	86.7% (13/15)	-
Naruka S et al. [66]	57.1% (8/14)	-
Jain K et al. [67]	60% (3/5)	-
Bhanuprasad K et al. [68]	NA	-
Desai EJ et al. [69]	-	-
Nasir \n et al. [70]	14.3% (1/7)	85.7% (6/7)
Gupta\s et al. [71]	NA	NA
Joshi S et al. [4]	NA	-
Pradhan P et al. [72]	55.6% (5/9)	-
Mehta RNM et al. [73]	57.7% (15/26)	-
Riad A et al. [74]	-	
Guzmán-Castro S et al. [75]	80% (4/5)	20% (1/5)
Seidel D et al. [76]	0% (0/7)	85.7% (6/7)
Gupta R et al. [77]	NA	-
Alfishawy M et al. [78]	71.4% (5/7)	0% (0/7)
Dave TV et al. [79]	NA	-
Selarka L et al. [80]	36.4% (4/11)	-
Avatef Fazeli M et al. [81]	-	-
Mishra Y et al. [82]	-	-

Sen M et al. [83]	NA	-
Pakdel F et al. [84]	NA	-
Y M. Reddy et al. [85]	0% (0/6)	-
R.Arora et al. [86]	NA	-
D.P Gupta et al. [87]	NA	-
M.Gautam et al. [88]	NA	-
R.M.Mehta et al. [89]	-	100% (4/4)
Y.M.Reddy et al. [90]	NA	-
S.P.Singh et al. [91]	0% (0/1)	-
M.Hada et al. [92]	NA	-
M. Kumar H et al. [93]	64.7% (11/17)	11.8% (2/17)
S. Bhandari et al. [94]	NA	NA
M Chouhan et al. [95]	NA	-
Y. Singh et al. [96]	22.2% (2/9)	11.1% (1/9)
S M Desai et al. [97]	NA	-
A. Kumari et al. [98]	66.7% (4/6)	-
S. Mitra et al. [99]	NA	-
A Ramaswami et al. [100]	NA	-
A.R. Joshi et al. [101]	NA	-
A. Patel et al. [102]	NA	NA
S Sharma et al. [103]	NA	-
R. Kant et al. [104]	NA	NA
C. Eker et al. [105]	40% (2/5)	-
A.K. Pandit et al. [106]	NA	-
S.F. Youssif et al. [107]	NA	-
A. Sekaran, et al. [108]	NA	-
R. R. Shabana et al. [109]	NA	-
A. K Patel et al. [110]	NA	NA
H. D.D. Martins et al. [111]	NA	-
S. Iqtadar et al. [112]	NA	-
A. Al Balushi et al. [113]	50% (3/6)	-
R. Soman et al. [114]	NA	-

NA: not available, - : no cerebral or pulmonary involvement
